# Supplementary material for: Human feeding biomechanics: performance, variation, and functional constraints
Source: PeerJ. 2016 Jul 26;4:e2242. doi: 10.7717/peerj.2242 (PMC4975005; doi:10.7717/peerj.2242)
Supplement: Supplemental Information 1 [file peerj-04-2242-s001.docx]

**SUPPLEMENTARY METHODS**

**Collection of human cortical bone mechanical properties**

The elastic (Young’s) modulus (*E*) and Poisson’s ratio (*v*) of craniofacial cortical bone in two-fresh frozen human crania (female, aged 22; male, aged 42) were quantified at 29 locations across the craniofacial skeletons (Table S1) by measuring their resistance to ultrasonic wave propagation (Peterson and Dechow, 2002; Schwartz-Dabney and Dechow, 2002; Wang and Dechow, 2006; Wang et al., 2006). For each cranium, cylindrical discs were cut from 29 locations across the cranial vault, zygomatic arch, circumorbital region, rostrum, alveolus, and palate on the left side of the cranium using a straight dental slow speed handpiece and 5.0 mm circular trephine bur attachment. The samples were removed under a ventilated hood while being continuously cooled by a water drip. Prior to removal, reference lines demarcating specimen orientation parallel to the occlusal plane were marked with a graphite pen at each of the 29 sites. Following specimen removal, cancellous bone on the inner surface of each cortical sample was removed using a water-cooled lathe.

Ultrasonic testing was performed at the Texas A&M University Baylor College of Dentistry. Prior to testing, specimen diameter, thickness, moist weight, and submerged weight were measured, and apparent densities in mg/cm^3^ were calculated based on Archimedes’ principle of buoyancy (Ashman et al., 1984). To quantify each specimen’s resistance to ultrasound wave propagation, bone samples were then mounted to a 4-inch rotary table allowing 360° rotation accurate to one tenth a degree. Pulse delays induced by the transmission of an ultrasonic wave, generated by piezoelectric transducers (5.0MHz V157-RM; Olympus NDT Inc, MA) powered by a Hewlett-Packard pulse generator (Model 8100) through the bone cylinder were measured at 10° intervals using a Tektronix TDS3032B digitizing oscilloscope. Delays were measured from 0° to 180° in a clockwise direction, with the graphite reference line representing 0°. Ultrasonic velocities at each of the 10° intervals were calculated by dividing the distance of wave transmission (i.e., diameter or thickness) by the measured pulse delay minus the standard system delay.

Specimen thickness, density, and ultrasonic velocity measurements were used in a MATHCAD program that calculated the elastic constants based on linear elastic wave theory and Hooke’s Law (Ashman et al., 1984). These include three elastic (Young’s) moduli (*E*_1_, *E*_2_, *E*_3_) and three Poisson’s ratios (*v*_12_, *v*_13_, *v*_23_). The Young’s modulus (*E*) of a material represents the slope of a stress-strain curve within its elastic range (i.e., before yield). It therefore represents a material’s ability to resist uniaxial stress. Subscripts for *E*_1_, *E*_2_, and *E*_3_ represent axes of stiffness through the thickness of the cortical plate, minimum stiffness, and maximum stiffness, respectively. Poisson’s ratio (*v*) represents a material’s resistance to deformation perpendicular to an applied load. In other words, it measures how much a material contracts when tensed or expands when compressed. For Poisson’s ratios, the first subscript represents the direction of the applied load, while the second subscript represents the tensile or compressive direction.

**Collection of *in vitro* bone strain**

We collected *in vitro* bone strain data from one cadaveric human cranium during simulations of P^3^ biting at Texas A&M University Baylor College of Dentistry for use in a validation analysis. In loading our specimen, we attempted to simulate muscle contraction by applying tension to the freed ends of the masseter and temporalis muscles, similar to recent analyses by Szwedowski, Fialkov & Whyne (2011) and Maloul et al. (2012). The 42 year old male specimen chosen for analysis was in good condition and it possessed all teeth with the exception of the central incisors and third molars. It was lacking any obvious craniofacial pathology with the exception of a small abscess near the right lateral incisor. To prepare the specimen for loading, the skin and superficial tissues were removed and a square window in the posterior cranial vault was cut using a Stryker autopsy saw. The brain was removed so that the specimen could be properly mounted and left to soak in a solution of alcohol and saline solution for 24 hours. It was then cleaned of remaining skin, cartilage, and soft tissue using scalpel and scissors, with the exception of the temporalis and masseter muscles. These muscles were cleaned and their bony mandibular insertions (i.e., coronoid process and mandibular ramus) were freed using a Dremel rotary saw. The temporalis inserts mainly to the coronoid process, but there is also a slip of muscle fibers that attaches to the anterior surface of the mandibular ramus. These temporalis fibers were cut, as was the attachment of the temporalis fascia along the superior edge of the zygomatic arch. The coronoid process and mandibular ramus were then used to pull the cranium into the loading apparatus during the collection of bone strain data (see below).

Five 0.25in diameter holes were drilled into the cranial base for mounting: four holes surrounding the foramen magnum and one hole drilled through the basiocciput. A 9×2.5×0.5in stainless steel mounting plate, aligned to the occlusal plane of the specimen, was then bolted to the cranium using stainless steel machine screws (#10) such that plate extended posteriorly. This allowed the specimen to be secured to a mounting apparatus constructed from stainless steel rods, spherical joints, and a large steel base designed to slide into an INSTRON 5567 loading machine (TestResources), which was used to align a steel rod to the tallest cusp on the left P^3^. The coronoid processes and mandibular rami were each secured to a 1/16in galvanized wire rope using machine screws (#5) and wire clamps. Each of the four wires ran through a set of pulleys and was attached to a disc weight (or weights) at the opposite end of the mounting apparatus. Each temporalis was attached to 12.5 pounds, while each masseter was attached to 10 pounds, for a total of 45 pounds of simulated muscle force (approximately 200N). This load was chosen because the mounting apparatus could not accommodate larger and/or additional weights and because of the risk of tearing muscle tissue when suspending the weights. Figure S1 illustrates the specimen and mounting apparatus during a simulated “bite” on the left P^3^.

Strain gages were affixed to various locations across the craniofacial skeleton just prior to loading. A total of 14 sites were chosen for gage placement. These sites correspond to those in Fig. S3 – Fig. S7. To prepare bone surfaces for gage placement, the periosteum at the 14 locations was first reflected and bone surfaces were polished with fine-grit sandpaper. Bone surfaces were degreased using n-Propyl bromide and then cleaned using phosphoric acid (M-Prep Conditioner A, Vishay Precision Group), followed by ammonia water (M-Prep Neutralizer). Three-element (45°) rosette strain gages (UFRA-1-11-3LT; Tokyo Sokki Kenkyujo Co., Ltd.) were bonded to the 14 locations using cyanoacrylate.

The cranium was subjected to three trials of simulated biting on the left P^3^. During muscle loading, a stainless steel rod with a 4.5mm circular tip was aligned normal to the tip of the tallest cusp and secured in place by applying a small load (~1-2N) using the loading machine. When suspending the weights, the mounting plate (and the attached cranium) was permitted to rotate about a horizontal axis located 125mm from the set of posterior-most mounting screws, thus allowing the cranium to be pulled up into the steel rod, generating stress and strain in the craniofacial skeleton. The weights were slowly lowered at a constant speed during loading, and any data from loadings with unusual results were discarded. This sometimes occurred if the weights were “dropped” too rapidly, causing a large spike in strain, or if a gage wire became caught on the mounting apparatus. Between trials, the weights were supported by a stand so that they were not continually being suspended, which would overstretch and/or tear the muscle tissue fibers.

During loading, raw strain data was measured by a series of synchronized PCD-300A sensor interfaces and recorded using DCS-300A strain data acquisition software (Kyowa Electronic Instruments). These raw strain data files were used to calculate minimum principal strain, maximum principal strain, and shear strain for each of the 14 locations using the strain data analyzer program DAS-200A (Kyowa Electronic Instruments). Principal strain orientations relative to the A-element of each rosette were also calculated. During and between loading trials, the specimen was kept moist by wrapping it in paper towels soaked with a solution of alcohol and saline. Photographs of each step of the analysis, including detailed images of gage positions, were collected so that it could be carefully replicated in the FEA described below.

**Creation of human cranial FEM for specimen-specific validation**

Prior to the collection of *in vitro* bone strain, the head was CT-scanned at Baylor’s Imaging Center and the digital images were used to generate a specimen-specific FEM following methods outlined above. Briefly, scans were used to generate a STL-formatted surface model using Mimics 16.0 which was refined in Geomagic Studio 2014. As with the specimens modeled in the analysis of variation, volumes of trabecular bone nested within the face were created using Geomagic Studio and measurements taken from the digital images. During this phase, a simple 9×2.5×0.5in rectangular volume representing the steel mounting plate and five 0.25in diameter cylindrical volumes representing the steel bolts were also constructed. Using measurements and images taking during the *in vitro* analysis, five 0.25in diameter holes were added to the model of the cranium, corresponding to the holes drilled in the specimen for mounting. Similarly, five 0.25in diameter holes were added to the volume representing the steel plate. The holes in both the cranium and the plate were created by copying and inverting the bolt volumes so that the five bolts and the five holes they passed through shared the same polygons, thus allowing the creation of one continuous model (Fig. S2). A solid FEM composed of composed 4-noded tetrahedral elements that make up the cranium, mounting plate, and mounting bolts, was generating from the surface model using the 3-Matic module of Mimics (Materialise N.V.). The mutual sharing of surfaces between the mounting bolts and the cranium and mounting plate created in Geomagic Studio allowed the meshing of one continuous solid, thus permitting forces to be transmitted throughout all components of the FEM, while allowing each volume to be assigned its own unique set of mechanical properties.

The solid FEM of the human cranium and mounting hardware was imported as a NAS file into Strand7 (Strand7 Pty Ltd), where material properties were assigned and a loading simulation mimicking the *in vitro* analysis was performed. The mechanical properties for cortical bone in the model were assigned based on samples harvested from various locations across the craniofacial skeleton in the 42 year old male specimen only (see Table S1). These properties were collected from the donor following completion of the loading analysis and were distributed throughout the cortical volume using the thermal diffusion technique described above. All remaining materials were modeled as homogeneous and isotropic. Trabecular bone and dental enamel were assigned properties identical to those assigned to the sample of seven variable human cranial FEMs (see above). The volumes representing the mounting plate and five mounting bolts were assigned default properties for stainless steel (UNS No. S30815) stored in the Strand7 program (elastic modulus = 200,000 MPa, Poisson’s ratio = 0.28).

**Muscle forces and constraints applied to the model under validation**

To replicate the action of pulling on the left and right temporalis and masseter muscles, plate elements covering the origins of the muscles were used to apply a superiorly directed force, with the model positioned such that the occlusal surfaces face superiorly, as in Fig. S2. Using Geomagic Studio 2014 and the images taken during the *in vitro* analysis as a guide, polygons covering the origins of the left and right temporalis and masseter were first selected from a surface model (STL) of the specimen. These polygons were copied and imported as plate elements into Strand7 where they were modeled as 3D membranes with a thickness of 0.001 mm and assigned the properties of cortical bone (an average value from the 29 sampled locations). Plate elements were then “zipped” to the surface of the solid model of the cranium. Vertically directed forces corresponding to the disc weights (plus ~60g for the wire clamps) were then applied to the muscle using a global face load distributed across the area of each muscle’s origin. Each temporalis origin was loaded with 56N of force, while each masseter was loaded with 45N, for a total of 202N of combined muscle force.

During muscle loading, the model was constrained at four nodes. Three nodes were chosen from the inferior surface of the mounting plate that were aligned in the same horizontal path and positioned approximately 125mm from the posterior mounting screws, corresponding to the point of rotation during the *in vitro* loading experiments. These nodes were constrained from translation but were permitted to rotate about a medio-lateral axis upon the application of muscle forces, as in the *in vitro* analysis. A fourth node at the tip of the tallest cusp of the left P^3^ was also constrained from translation, replicating the “bite point” from the *in vitro* analysis. This induced deformation and generated strains in the craniofacial skeleton when loaded with muscle forces.

**Sensitivity Analysis: modeling of muscle forces and constraints**

We conducted a sensitivity analysis to assess the impact of some differences in methodology between our study and a study performed by Wroe et al. (2010), who examined the feeding biomechanics of modern humans, other hominoids, and fossil australopiths using finite element analysis (FEA). Some of the main differences between our analysis and the analysis performed by Wroe et al. (2010) pertain to the manner in which the jaw adductor muscle forces were modeled and how the constraints were applied. These differences potentially explain the discrepancy in results related to the strength of the human craniofacial skeleton between the two studies. Specifically, we find the human face to be weaker relative to that of chimpanzees, whereas Wroe et al. (2010) suggest that the human face may be relatively strong.

Wroe et al. (2010) loaded their models with muscles modeled as straight pre-tensioned beam elements extending from points on the cranium to the mandible within the Strand7 FEA software. We used a program (Boneload) to apply both normal and tangential tractions over entire muscle origins, with muscle force vectors oriented toward points that were defined by muscle insertion sites. This technique accounts for the added torque produced when muscles wrap around curved bone surfaces (Grosse et al., 2007). A further difference between the two studies is that Wroe et al. (2010) included the posterior temporalis in their analysis, whereas we only consider the anterior temporalis. Smith et al. (2015a) conjecture that some of the beam elements representing the posterior fibers of temporalis of at least one of their FEMs (*Paranthropus boisei* specimen OH5) could have potentially passed below the TMJ in the analysis by Wroe et al. (2010), which would have exerted torques that oppose those of the other muscle beams.

In addition to differences in muscle modeling, there were differences in the way constraints were applied to the models analyzed by Wroe et al. (2010) and those analyzed here. In our analysis, FEMs were constrained at a single node against translation in all directions at the working-side TMJ, while the balancing-side TMJ was constrained in the superoinferior and anteroposterior directions (Strait et al., 2009; Smith et al., 2015a,b). The biting tooth (either P^3^ or M^2^) was also constrained in the superoinferior direction at a node in the center of the occlusal surface. Wroe et al. (2010) instead constructed a hinge at each TMJ using a system of beam elements and rigid links. The advantage of this is that the cranium and mandible can be analyzed together in a single analysis. In their study, Wroe et al. (2010) also constrained the biting tooth at a center node but in all directions. These authors also tessellated the articular surfaces of both TMJs, as well as the occlusal surface of the biting tooth, using very stiff beam elements in order to decrease local concentrations of stress and strain.

We analyzed P^3^ biting in one of our human FEMs (GRGL) with constraints identical to those in Wroe et al. (2010) and with the anterior temporalis, superficial masseter, deep masseter, and medial pterygoid muscles modeled as straight pre-tensioned beam elements using the same forces applied to the ALL-HUM models. For this analysis, it was necessary to generate a solid FEM of the mandible, which we modeled as a solid cortical structure. We also analyzed a second model in this way, but with the addition of muscle beams representing the posterior temporalis. As with the other ALL-HUM muscle forces, the posterior temporalis force was based on muscle physiological cross-sectional area (PCSA) data reported by van Eijden, Korfagen & Brugman (1997), corrected to account for pennation and differences in gape during fixation using formulae from Taylor & Vinyard (2013). Using our original muscle origin and insertion plates as a guide, beam elements were extended from the cranium to the mandible (Fig. S8) and were assigned pre-tension forces calculated by dividing the total force by the number of beams for each muscle group (Table S4). A TMJ “hinge” connecting the mandibular condyle and glenoid fossa was constructed on the left and right sides using a system of rigid links and beam elements that were assigned the properties of steel (Young’s modulus = 200 GPa, Poisson’s ratio = 0.25). The occlusal surface of the left P^3^ was fully fixed by a central node in all directions. Both the occlusal surface of the fixed tooth and the articular surfaces at the TMJs were tessellated using beam elements that were also assigned the properties of steel.

We found that the two FEMs analyzed following the methods of Wroe et al. (2010) generated a pattern of von Mises strain magnitudes very similar to our original model (Fig. S9). Data on von Mises strain collected from identical bricks at 14 sampled locations reveal that these differences in methodology only result in small differences in strain magnitude at most locations across the craniofacial skeleton (Fig. S10), with the largest differences found at locations 8 (working zygomatic root), 12 (working nasal margin), and 14 (balancing zygomatic body). There was no clear pattern to these differences; our original “boneloaded” model experienced strain magnitudes that were either very similar to, greater than, or less than the “beamed” variants depending on location. Interestingly, the model that included beams for the posterior temporalis was found to exhibit strain magnitudes that were lower than our original “boneloaded” model at 6 locations and lower than the “beamed” model lacking a posterior temporalis at 4 locations despite being loaded with an extra 130.23 N on both sides of the head. When applying beams for the posterior temporalis to this model, one or two of the 20 beams representing this muscle did indeed pass below the axis of rotation at the TMJ hinge on both sides of the head, so the torques of these beams opposed those of the other muscle beams (see Smith et al., 2015a). However, this amounted to only a very small proportion of the total muscle force. Therefore, even if the same occurred in the Wroe et al. (2010) study, differences in the modeling of muscle loads and constraints are unlikely to explain the observed differences in results between the two sets of analyses. Instead, these differences are more likely to be related to methodological differences in muscle force scaling (see Main Text).
