# Supplementary material for: Human feeding biomechanics: performance, variation, and functional constraints
Source: PeerJ. 2016 Jul 26;4:e2242. doi: 10.7717/peerj.2242 (PMC4975005; doi:10.7717/peerj.2242)
Supplement: Supplemental Information 2 — E3 and v23 refer to the elastic (Young’s) modulus and Poisson’s ratio in the axis of maximum stiffness, respectively. For modulus, factor and temperature data were used to distribute regionally variation mechanical properties throughout each of the ALL-HUM models (see Main Text). [file peerj-04-2242-s002.docx]

| **No.** | **Site** | ***E*3** | | | | |  | ***v*23** | |
| --- | --- | --- | --- | --- | --- | --- | --- | --- | --- |
|  |  | **Male** | **Female** | **Avg** | **Factor^1^** | **Temp^1^** |  | **Male** | **Female** |
| **1** | Posterior vault | 20.017 | 20.354 | 20.186 | 0.876 | 43.813 |  | 0.286 | 0.303 |
| **2** | Sup-posterior vault | 21.868 | 18.387 | 20.128 | 0.874 | 43.687 |  | 0.332 | 0.295 |
| **3** | Posterior parietal | 26.588 | 20.818 | 23.703 | 1.029 | 51.448 |  | 0.293 | 0.302 |
| **4** | Anterior parietal | 23.321 | 18.185 | 20.753 | 0.901 | 45.045 |  | 0.315 | 0.319 |
| **5** | Sup-anterior vault | 22.158 | 16.948 | 19.553 | 0.849 | 42.440 |  | 0.260 | 0.303 |
| **6** | Anterior vault | 23.893 | 18.823 | 21.358 | 0.927 | 46.358 |  | 0.255 | 0.304 |
| **7** | Mid dorsal orbital | 21.407 | 23.337 | 22.372 | 0.971 | 48.559 |  | 0.262 | 0.315 |
| **8** | Lateral dorsal orbital | 19.353 | 18.975 | 19.164 | 0.832 | 41.596 |  | 0.239 | 0.290 |
| **9** | Posterior temporal | 19.701 | 16.337 | 18.019 | 0.782 | 39.111 |  | 0.288 | 0.337 |
| **10** | Mid temporal | 24.618 | 16.988 | 20.803 | 0.903 | 45.153 |  | 0.312 | 0.309 |
| **11** | Posterior zygo-arch | 22.093 | 20.760 | 21.427 | 0.930 | 46.507 |  | 0.288 | 0.320 |
| **12** | Anterior temporal | 20.095 | 20.919 | 20.507 | 0.890 | 44.511 |  | 0.235 | 0.294 |
| **13** | Mid zygo-arch | 27.208 | 18.855 | 23.032 | 0.999 | 49.990 |  | 0.266 | 0.289 |
| **14** | Zygoma | 16.467 | 19.577 | 18.022 | 0.782 | 39.117 |  | 0.292 | 0.303 |
| **15** | Postorbital bar | 21.289 | 25.814 | 23.552 | 1.022 | 51.119 |  | 0.297 | 0.258 |
| **16** | Nasal margin | 26.423 | 19.649 | 23.036 | 1.000 | 50.000 |  | 0.245 | 0.316 |
| **17** | Infraorbital | 21.845 | 18.169 | 20.007 | 0.869 | 43.426 |  | 0.325 | 0.242 |
| **18** | Zygomatic root | 26.355 | 24.686 | 25.521 | 1.108 | 55.393 |  | 0.295 | 0.293 |
| **19** | Third molar | 19.538 | 21.888 | 20.713 | 0.899 | 44.958 |  | 0.343 | 0.290 |
| **20** | Molar 1/2 | 20.834 | 23.586 | 22.210 | 0.964 | 48.207 |  | 0.298 | 0.311 |
| **21** | Premolar 4 | 21.431 | 21.255 | 21.343 | 0.927 | 46.325 |  | 0.336 | 0.336 |
| **22** | Incisor 2 | 20.048 | 16.938 | 18.493 | 0.803 | 40.139 |  | 0.257 | 0.282 |
| **23** | Lateral orbital wall | 19.059 | 18.330 | 18.695 | 0.812 | 40.577 |  | 0.347 | 0.252 |
| **24** | Inferior orbital wall^1^ | 18.545 | 18.545 | 18.545 | 0.805 | 40.252 |  | 0.333 | 0.333 |
| **25** | Medial orbital wall | 20.562 | 15.283 | 17.923 | 0.778 | 38.901 |  | 0.255 | 0.321 |
| **26** | Superior orbital wall^2^ | 18.957 | 18.957 | 18.957 | 0.823 | 41.146 |  | 0.257 | 0.257 |
| **27** | Antero-lateral palate | 21.994 | 18.125 | 20.060 | 0.871 | 43.539 |  | 0.247 | 0.276 |
| **28** | Postero-lateral palate | 19.408 | 20.510 | 19.959 | 0.866 | 43.321 |  | 0.340 | 0.217 |
| **29** | Dorsal interorbital | 17.908 | 21.620 | 19.764 | 0.858 | 42.898 |  | 0.331 | 0.299 |

^1^Values calculated based on the average *E*3.

^2^The cortical specimen collected from the female specimen from this site was unsuitable for property measurement, so the male value was substituted instead.

^3^The cortical specimen collected from the male specimen from this site was unsuitable for property measurement, so the female value was substituted instead.
