# Supplementary material for: Human feeding biomechanics: performance, variation, and functional constraints
Source: PeerJ. 2016 Jul 26;4:e2242. doi: 10.7717/peerj.2242 (PMC4975005; doi:10.7717/peerj.2242)
Supplement: Supplemental Information 3 — Maximum principal strain (MaxPrin), minimum principal strain (MinPrin), strain mode (Mode), maximum shear strain (Shear), von Mises strain, and strain energy density (SED) generated during simulated premolar (P3) biting in the ALL-HUM variants of “extreme” and “average” modern human cranial FEMs. Site numbers follow Fig. 4. [file peerj-04-2242-s003.docx]

| **Site** | **Model** | **MaxPrin (με)** | **MinPrin (με)** | **Mode** | **Shear (με)** | **von Mises (με)** | **SED**  **(J/mm^3^)** |
| --- | --- | --- | --- | --- | --- | --- | --- |
| **1** | GRGL | 27 | -61 | 0.44 | 88 | 79 | 0.05 |
|  | BERG | 26 | -41 | 0.63 | 67 | 60 | 0.02 |
|  | KSAN1 | 23 | -29 | 0.80 | 52 | 45 | 0.01 |
|  | KSAN2 | 18 | -38 | 0.47 | 56 | 50 | 0.02 |
|  | MALP | 38 | -57 | 0.67 | 95 | 85 | 0.04 |
|  | TIGA | 11 | -33 | 0.33 | 44 | 44 | 0.01 |
|  | WAFR | 61 | -24 | 2.57 | 84 | 79 | 0.04 |
| **2** | GRGL | 56 | -18 | 3.07 | 74 | 72 | 0.04 |
|  | BERG | 29 | -15 | 1.98 | 44 | 41 | 0.01 |
|  | KSAN1 | 34 | -12 | 2.86 | 46 | 44 | 0.01 |
|  | KSAN2 | 35 | -14 | 2.50 | 49 | 46 | 0.01 |
|  | MALP | 35 | -13 | 2.74 | 48 | 45 | 0.01 |
|  | TIGA | 32 | -10 | 3.13 | 42 | 40 | 0.01 |
|  | WAFR | 55 | -30 | 1.80 | 85 | 76 | 0.04 |
| **3** | GRGL | 59 | -19 | 3.11 | 78 | 75 | 0.04 |
|  | BERG | 49 | -25 | 2.01 | 74 | 68 | 0.03 |
|  | KSAN1 | 35 | -15 | 2.33 | 50 | 47 | 0.01 |
|  | KSAN2 | 32 | -13 | 2.46 | 45 | 42 | 0.01 |
|  | MALP | 39 | -13 | 3.11 | 52 | 50 | 0.02 |
|  | TIGA | 43 | -14 | 3.02 | 57 | 55 | 0.02 |
|  | WAFR | 58 | -20 | 2.97 | 78 | 74 | 0.04 |
| **4** | GRGL | 234 | -140 | 1.67 | 374 | 335 | 0.69 |
|  | BERG | 203 | -116 | 1.75 | 318 | 286 | 0.51 |
|  | KSAN1 | 174 | -128 | 1.36 | 302 | 265 | 0.42 |
|  | KSAN2 | 190 | -170 | 1.11 | 360 | 312 | 0.58 |
|  | MALP | 156 | -120 | 1.29 | 276 | 241 | 0.35 |
|  | TIGA | 162 | -81 | 2.00 | 243 | 223 | 0.32 |
|  | WAFR | 219 | -122 | 1.80 | 341 | 304 | 0.60 |
| **5** | GRGL | 296 | -121 | 2.45 | 416 | 392 | 1.02 |
|  | BERG | 254 | -112 | 2.27 | 366 | 340 | 0.76 |
|  | KSAN1 | 207 | -119 | 1.74 | 326 | 295 | 0.54 |
|  | KSAN2 | 267 | -141 | 1.89 | 408 | 369 | 0.87 |
|  | MALP | 224 | -110 | 2.03 | 334 | 306 | 0.60 |
|  | TIGA | 203 | -95 | 2.14 | 298 | 276 | 0.49 |
|  | WAFR | 270 | -135 | 2.00 | 405 | 374 | 0.88 |
| **6** | GRGL | 73 | -255 | 0.29 | 328 | 319 | 0.75 |
|  | BERG | 78 | -239 | 0.33 | 317 | 308 | 0.66 |
|  | KSAN1 | 85 | -187 | 0.45 | 272 | 249 | 0.42 |
|  | KSAN2 | 129 | -237 | 0.54 | 366 | 330 | 0.69 |
|  | MALP | 79 | -195 | 0.41 | 274 | 255 | 0.44 |
|  | TIGA | 84 | -202 | 0.42 | 286 | 266 | 0.48 |
|  | WAFR | 103 | -244 | 0.42 | 347 | 323 | 0.70 |
| **7** | GRGL | 101 | -217 | 0.47 | 318 | 290 | 0.56 |
|  | BERG | 106 | -257 | 0.41 | 363 | 341 | 0.77 |
|  | KSAN1 | 84 | -199 | 0.42 | 283 | 264 | 0.47 |
|  | KSAN2 | 76 | -234 | 0.32 | 310 | 304 | 0.63 |
|  | MALP | 106 | -225 | 0.47 | 331 | 303 | 0.60 |
|  | TIGA | 87 | -218 | 0.40 | 305 | 291 | 0.55 |
|  | WAFR | 99 | -241 | 0.41 | 340 | 316 | 0.68 |
| **8** | GRGL | 60 | -195 | 0.31 | 255 | 247 | 0.44 |
|  | BERG | 81 | -242 | 0.33 | 323 | 297 | 0.71 |
|  | KSAN1 | 87 | -246 | 0.35 | 333 | 312 | 0.71 |
|  | KSAN2 | 106 | -238 | 0.45 | 344 | 305 | 0.71 |
|  | MALP | 78 | -238 | 0.33 | 316 | 291 | 0.69 |
|  | TIGA | 92 | -243 | 0.38 | 335 | 311 | 0.70 |
|  | WAFR | 111 | -300 | 0.37 | 411 | 376 | 1.08 |
| **9** | GRGL | 27 | -72 | 0.38 | 99 | 96 | 0.06 |
|  | BERG | 30 | -51 | 0.59 | 81 | 72 | 0.03 |
|  | KSAN1 | 36 | -68 | 0.53 | 104 | 94 | 0.06 |
|  | KSAN2 | 17 | -48 | 0.36 | 66 | 64 | 0.03 |
|  | MALP | 27 | -57 | 0.47 | 83 | 73 | 0.04 |
|  | TIGA | 26 | -68 | 0.38 | 94 | 86 | 0.06 |
|  | WAFR | 27 | -38 | 0.71 | 65 | 57 | 0.02 |
| **10** | GRGL | 106 | -61 | 1.75 | 167 | 149 | 0.14 |
|  | BERG | 92 | -72 | 1.27 | 164 | 143 | 0.12 |
|  | KSAN1 | 99 | -87 | 1.14 | 186 | 162 | 0.16 |
|  | KSAN2 | 80 | -55 | 1.47 | 135 | 117 | 0.09 |
|  | MALP | 103 | -46 | 2.26 | 149 | 138 | 0.13 |
|  | TIGA | 78 | -117 | 0.67 | 195 | 170 | 0.18 |
|  | WAFR | 98 | -65 | 1.51 | 164 | 144 | 0.13 |
| **11** | GRGL | 68 | -35 | 1.96 | 103 | 93 | 0.06 |
|  | BERG | 59 | -18 | 3.28 | 78 | 77 | 0.04 |
|  | KSAN1 | 51 | -38 | 1.34 | 88 | 78 | 0.04 |
|  | KSAN2 | 30 | -36 | 0.84 | 66 | 58 | 0.02 |
|  | MALP | 46 | -23 | 2.00 | 70 | 65 | 0.03 |
|  | TIGA | 64 | -54 | 1.19 | 118 | 103 | 0.06 |
|  | WAFR | 64 | -55 | 1.16 | 120 | 104 | 0.06 |
| **12** | GRGL | 150 | -490 | 0.31 | 640 | 625 | 2.77 |
|  | BERG | 85 | -283 | 0.30 | 368 | 362 | 0.92 |
|  | KSAN1 | 119 | -379 | 0.31 | 498 | 480 | 1.66 |
|  | KSAN2 | 277 | -639 | 0.43 | 916 | 847 | 4.83 |
|  | MALP | 171 | -484 | 0.35 | 655 | 623 | 2.72 |
|  | TIGA | 76 | -225 | 0.34 | 301 | 288 | 0.59 |
|  | WAFR | 282 | -619 | 0.46 | 901 | 835 | 4.54 |
| **13** | GRGL | 42 | -13 | 3.09 | 55 | 54 | 0.02 |
|  | BERG | 39 | -16 | 2.53 | 55 | 53 | 0.02 |
|  | KSAN1 | 69 | -33 | 2.12 | 101 | 92 | 0.06 |
|  | KSAN2 | 57 | -33 | 1.73 | 90 | 80 | 0.04 |
|  | MALP | 36 | -20 | 1.79 | 57 | 51 | 0.02 |
|  | TIGA | 69 | -20 | 3.45 | 89 | 86 | 0.05 |
|  | WAFR | 41 | -26 | 1.61 | 67 | 60 | 0.02 |
| **14** | GRGL | 33 | -17 | 1.94 | 50 | 43 | 0.02 |
|  | BERG | 13 | -10 | 1.36 | 23 | 20 | 0.00 |
|  | KSAN1 | 27 | -15 | 1.85 | 41 | 36 | 0.01 |
|  | KSAN2 | 35 | -13 | 2.69 | 48 | 44 | 0.01 |
|  | MALP | 43 | -18 | 2.37 | 61 | 53 | 0.02 |
|  | TIGA | 51 | -21 | 2.43 | 72 | 64 | 0.03 |
|  | WAFR | 34 | -16 | 2.07 | 50 | 46 | 0.01 |
