# Supplementary material for: Human feeding biomechanics: performance, variation, and functional constraints
Source: PeerJ. 2016 Jul 26;4:e2242. doi: 10.7717/peerj.2242 (PMC4975005; doi:10.7717/peerj.2242)
Supplement: Supplemental Information 4 — Maximum principal strain (MaxPrin), minimum principal strain (MinPrin), strain mode (Mode), maximum shear strain (Shear), von Mises strain, and strain energy density (SED) generated during simulated molar (M2) biting in the ALL-HUM variants of “extreme” and “average” modern human cranial FEMs. Site numbers follow Fig. 4. [file peerj-04-2242-s004.docx]

| **Site** | **Model** | **MaxPrin (με)** | **MinPrin (με)** | **Mode** | **Shear (με)** | **von Mises (με)** | **SED**  **(J/mm^3^)** |
| --- | --- | --- | --- | --- | --- | --- | --- |
| **1** | GRGL | 19 | -43 | 0.44 | 62 | 58 | 0.02 |
|  | BERG | 31 | -25 | 1.23 | 56 | 49 | 0.01 |
|  | KSAN1 | 25 | -20 | 1.22 | 45 | 39 | 0.01 |
|  | KSAN2 | 16 | -28 | 0.57 | 44 | 39 | 0.01 |
|  | MALP | 33 | -31 | 1.09 | 64 | 55 | 0.02 |
|  | TIGA | 17 | -24 | 0.71 | 41 | 36 | 0.01 |
|  | WAFR | 49 | -21 | 2.34 | 70 | 65 | 0.03 |
| **2** | GRGL | 34 | -10 | 3.24 | 44 | 43 | 0.01 |
|  | BERG | 18 | -10 | 1.87 | 28 | 25 | 0.00 |
|  | KSAN1 | 26 | -10 | 2.60 | 36 | 35 | 0.01 |
|  | KSAN2 | 29 | -11 | 2.64 | 40 | 38 | 0.01 |
|  | MALP | 37 | -12 | 3.03 | 49 | 47 | 0.02 |
|  | TIGA | 20 | -8 | 2.64 | 28 | 25 | 0.01 |
|  | WAFR | 38 | -24 | 1.59 | 62 | 55 | 0.02 |
| **3** | GRGL | 60 | -19 | 3.16 | 79 | 74 | 0.04 |
|  | BERG | 51 | -21 | 2.40 | 73 | 70 | 0.03 |
|  | KSAN1 | 31 | -12 | 2.58 | 43 | 40 | 0.01 |
|  | KSAN2 | 33 | -12 | 2.75 | 45 | 44 | 0.01 |
|  | MALP | 40 | -13 | 3.09 | 52 | 50 | 0.02 |
|  | TIGA | 48 | -14 | 3.55 | 62 | 61 | 0.03 |
|  | WAFR | 58 | -19 | 3.13 | 77 | 73 | 0.04 |
| **4** | GRGL | 131 | -100 | 1.32 | 231 | 202 | 0.25 |
|  | BERG | 112 | -92 | 1.21 | 204 | 177 | 0.19 |
|  | KSAN1 | 129 | -118 | 1.09 | 248 | 214 | 0.27 |
|  | KSAN2 | 68 | -116 | 0.58 | 184 | 166 | 0.17 |
|  | MALP | 44 | -71 | 0.62 | 116 | 104 | 0.07 |
|  | TIGA | 113 | -62 | 1.84 | 175 | 159 | 0.16 |
|  | WAFR | 144 | -88 | 1.64 | 232 | 204 | 0.27 |
| **5** | GRGL | 287 | -107 | 2.69 | 393 | 375 | 0.95 |
|  | BERG | 235 | -96 | 2.44 | 331 | 312 | 0.65 |
|  | KSAN1 | 209 | -109 | 1.92 | 318 | 291 | 0.53 |
|  | KSAN2 | 258 | -130 | 1.98 | 388 | 354 | 0.81 |
|  | MALP | 229 | -103 | 2.22 | 332 | 307 | 0.62 |
|  | TIGA | 209 | -91 | 2.30 | 300 | 280 | 0.51 |
|  | WAFR | 271 | -131 | 2.07 | 402 | 374 | 0.88 |
| **6** | GRGL | 70 | -233 | 0.30 | 303 | 290 | 0.63 |
|  | BERG | 71 | -219 | 0.32 | 290 | 280 | 0.56 |
|  | KSAN1 | 78 | -170 | 0.46 | 248 | 227 | 0.35 |
|  | KSAN2 | 109 | -197 | 0.55 | 306 | 276 | 0.48 |
|  | MALP | 75 | -169 | 0.44 | 244 | 225 | 0.34 |
|  | TIGA | 81 | -198 | 0.41 | 279 | 260 | 0.46 |
|  | WAFR | 96 | -236 | 0.41 | 332 | 312 | 0.65 |
| **7** | GRGL | 100 | -212 | 0.47 | 312 | 285 | 0.54 |
|  | BERG | 97 | -250 | 0.39 | 347 | 329 | 0.73 |
|  | KSAN1 | 103 | -229 | 0.45 | 332 | 305 | 0.62 |
|  | KSAN2 | 74 | -223 | 0.33 | 297 | 291 | 0.57 |
|  | MALP | 103 | -217 | 0.47 | 320 | 293 | 0.56 |
|  | TIGA | 86 | -211 | 0.41 | 297 | 283 | 0.52 |
|  | WAFR | 96 | -231 | 0.42 | 327 | 304 | 0.63 |
| **8** | GRGL | 130 | -366 | 0.36 | 496 | 466 | 1.57 |
|  | BERG | 123 | -412 | 0.30 | 534 | 524 | 1.96 |
|  | KSAN1 | 107 | -327 | 0.33 | 434 | 416 | 1.25 |
|  | KSAN2 | 154 | -388 | 0.40 | 543 | 497 | 1.80 |
|  | MALP | 182 | -364 | 0.50 | 546 | 450 | 1.65 |
|  | TIGA | 111 | -289 | 0.38 | 400 | 372 | 0.98 |
|  | WAFR | 164 | -405 | 0.40 | 569 | 514 | 2.00 |
| **9** | GRGL | 33 | -87 | 0.38 | 120 | 111 | 0.09 |
|  | BERG | 29 | -75 | 0.39 | 104 | 97 | 0.07 |
|  | KSAN1 | 28 | -67 | 0.41 | 95 | 89 | 0.05 |
|  | KSAN2 | 22 | -73 | 0.30 | 96 | 91 | 0.06 |
|  | MALP | 31 | -69 | 0.44 | 99 | 87 | 0.06 |
|  | TIGA | 28 | -74 | 0.38 | 102 | 93 | 0.07 |
|  | WAFR | 32 | -68 | 0.47 | 100 | 90 | 0.06 |
| **10** | GRGL | 138 | -87 | 1.58 | 226 | 200 | 0.25 |
|  | BERG | 112 | -93 | 1.20 | 205 | 178 | 0.19 |
|  | KSAN1 | 78 | -63 | 1.24 | 141 | 122 | 0.09 |
|  | KSAN2 | 92 | -58 | 1.60 | 150 | 130 | 0.12 |
|  | MALP | 66 | -57 | 1.17 | 123 | 106 | 0.07 |
|  | TIGA | 81 | -96 | 0.84 | 177 | 154 | 0.14 |
|  | WAFR | 43 | -73 | 0.59 | 116 | 104 | 0.07 |
| **11** | GRGL | 53 | -28 | 1.93 | 81 | 73 | 0.03 |
|  | BERG | 40 | -13 | 3.10 | 53 | 51 | 0.02 |
|  | KSAN1 | 38 | -25 | 1.54 | 63 | 57 | 0.02 |
|  | KSAN2 | 24 | -25 | 0.96 | 48 | 42 | 0.01 |
|  | MALP | 33 | -17 | 1.91 | 50 | 46 | 0.01 |
|  | TIGA | 52 | -43 | 1.21 | 95 | 83 | 0.04 |
|  | WAFR | 46 | -45 | 1.02 | 90 | 78 | 0.04 |
| **12** | GRGL | 107 | -117 | 0.91 | 224 | 194 | 0.22 |
|  | BERG | 84 | -130 | 0.65 | 215 | 190 | 0.22 |
|  | KSAN1 | 50 | -92 | 0.55 | 142 | 128 | 0.10 |
|  | KSAN2 | 188 | -192 | 0.98 | 380 | 329 | 0.64 |
|  | MALP | 90 | -107 | 0.84 | 198 | 172 | 0.18 |
|  | TIGA | 37 | -80 | 0.46 | 116 | 106 | 0.08 |
|  | WAFR | 146 | -188 | 0.78 | 334 | 291 | 0.51 |
| **13** | GRGL | 112 | -50 | 2.23 | 162 | 151 | 0.15 |
|  | BERG | 78 | -37 | 2.12 | 115 | 107 | 0.07 |
|  | KSAN1 | 119 | -58 | 2.05 | 177 | 161 | 0.17 |
|  | KSAN2 | 105 | -42 | 2.51 | 147 | 139 | 0.13 |
|  | MALP | 89 | -50 | 1.77 | 139 | 125 | 0.10 |
|  | TIGA | 94 | -34 | 2.76 | 128 | 123 | 0.10 |
|  | WAFR | 97 | -59 | 1.64 | 156 | 139 | 0.12 |
| **14** | GRGL | 37 | -20 | 1.85 | 57 | 49 | 0.02 |
|  | BERG | 11 | -9 | 1.13 | 20 | 18 | 0.00 |
|  | KSAN1 | 29 | -16 | 1.86 | 45 | 39 | 0.01 |
|  | KSAN2 | 31 | -13 | 2.38 | 44 | 39 | 0.01 |
|  | MALP | 41 | -19 | 2.16 | 61 | 53 | 0.02 |
|  | TIGA | 56 | -22 | 2.55 | 78 | 70 | 0.04 |
|  | WAFR | 33 | -17 | 1.92 | 50 | 46 | 0.01 |
