# Supplementary material for: Human feeding biomechanics: performance, variation, and functional constraints
Source: PeerJ. 2016 Jul 26;4:e2242. doi: 10.7717/peerj.2242 (PMC4975005; doi:10.7717/peerj.2242)
Supplement: Supplemental Information 5 — Total muscle forces, beam count, and force per beam for each muscle group assigned to the GRGL model in the sensitivity analysis. Forces are in Newtons (N). [file peerj-04-2242-s005.docx]

| **Muscle** | **Total force** | **Beam count** | **Force per beam** |
| --- | --- | --- | --- |
| Anterior temporalis | 181.55 | 30 | 6.052 |
| Posterior temporalis | 130.23 | 20 | 6.512 |
| Superficial masseter | 148.66 | 18 | 8.259 |
| Deep masseter | 75.34 | 13 | 5.795 |
| Medial pterygoid | 153.60 | 17 | 9.035 |
