# Supplementary figures and images for: Human feeding biomechanics: performance, variation, and functional constraints
Source: PeerJ. 2016 Jul 26;4:e2242. doi: 10.7717/peerj.2242 (PMC4975005; doi:10.7717/peerj.2242)

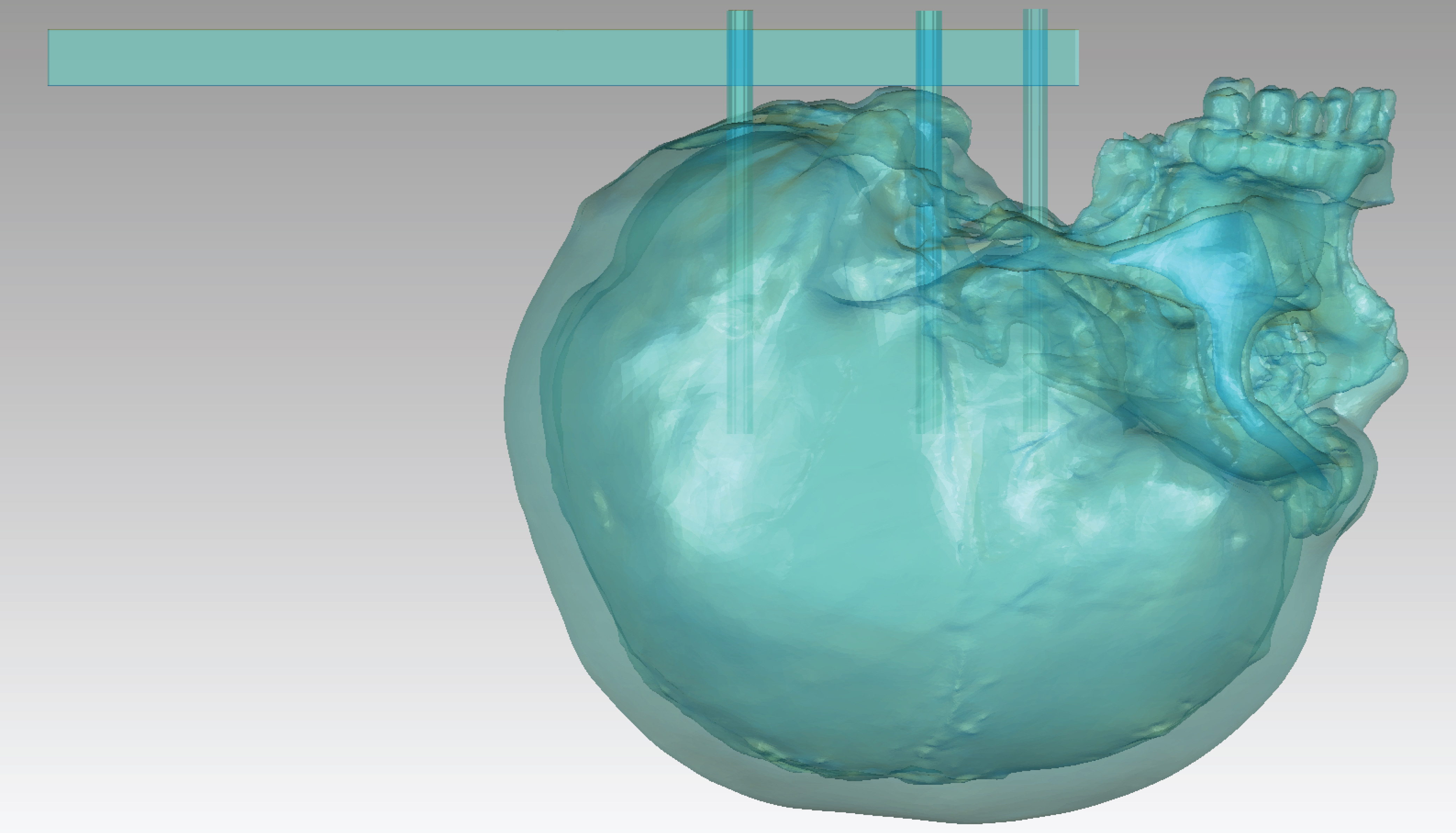

Supplement: Supplemental Information 7 — The surface model is shown in the position it was constrained during muscle loading, as in Fig. S1. [file peerj-04-2242-s007.jpg]

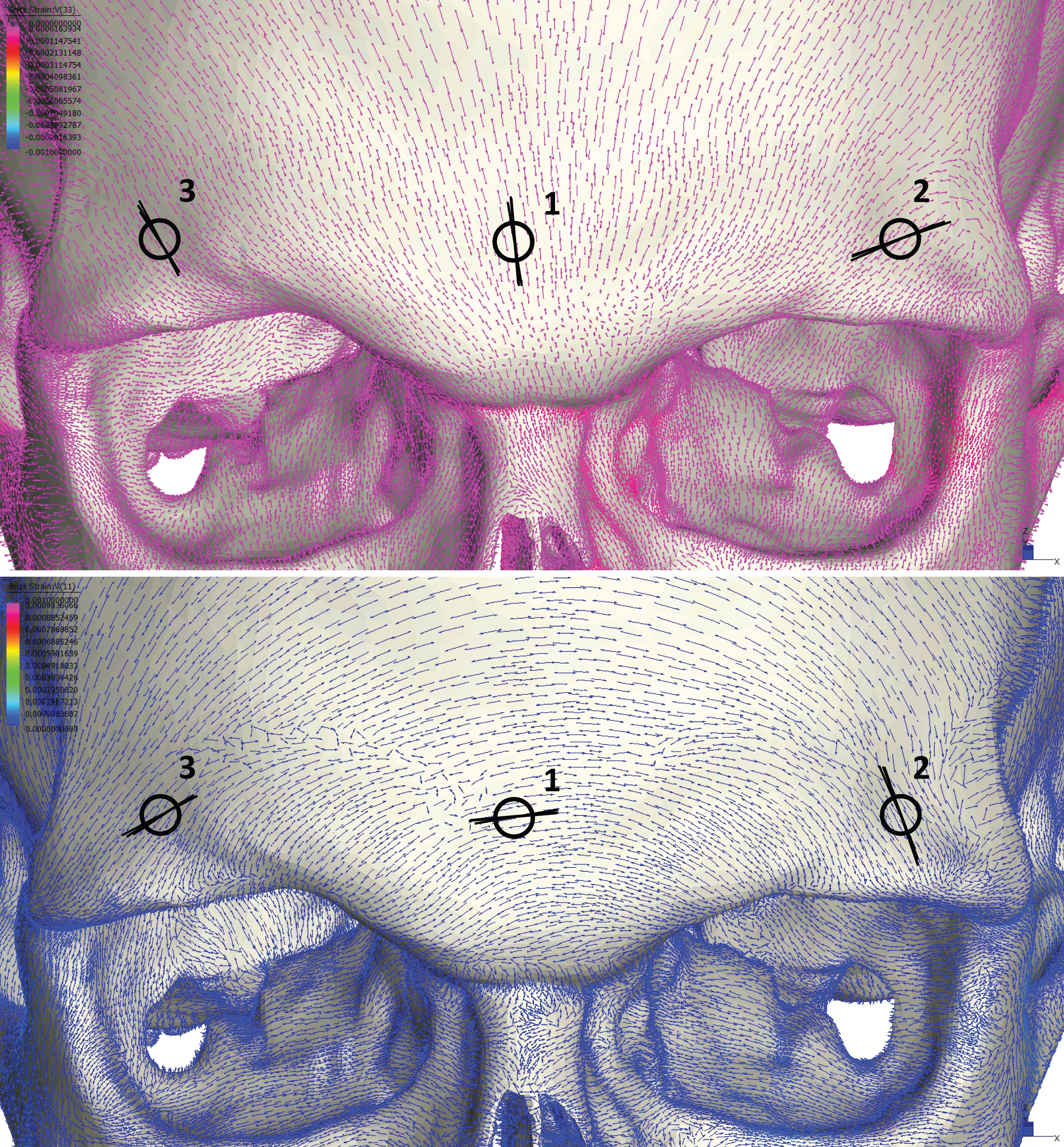

Supplement: Supplemental Information 8 — Purple lines represent the orientation of minimum principal strain (compression), which is 90° to orientation of maximum principal strain. Black circles represent location of strain gages at the dorsal interorbital (site 1), working-side dorsal orbital (site 2), and balancing-side dorsal orbital (site 3) during in vitro bone strain analysis. Three lines through each gage correspond to the orientation of principal strains during the in vitro loading analysis which were recorded in degrees relative to the A element of the gage. [file peerj-04-2242-s008.jpg]

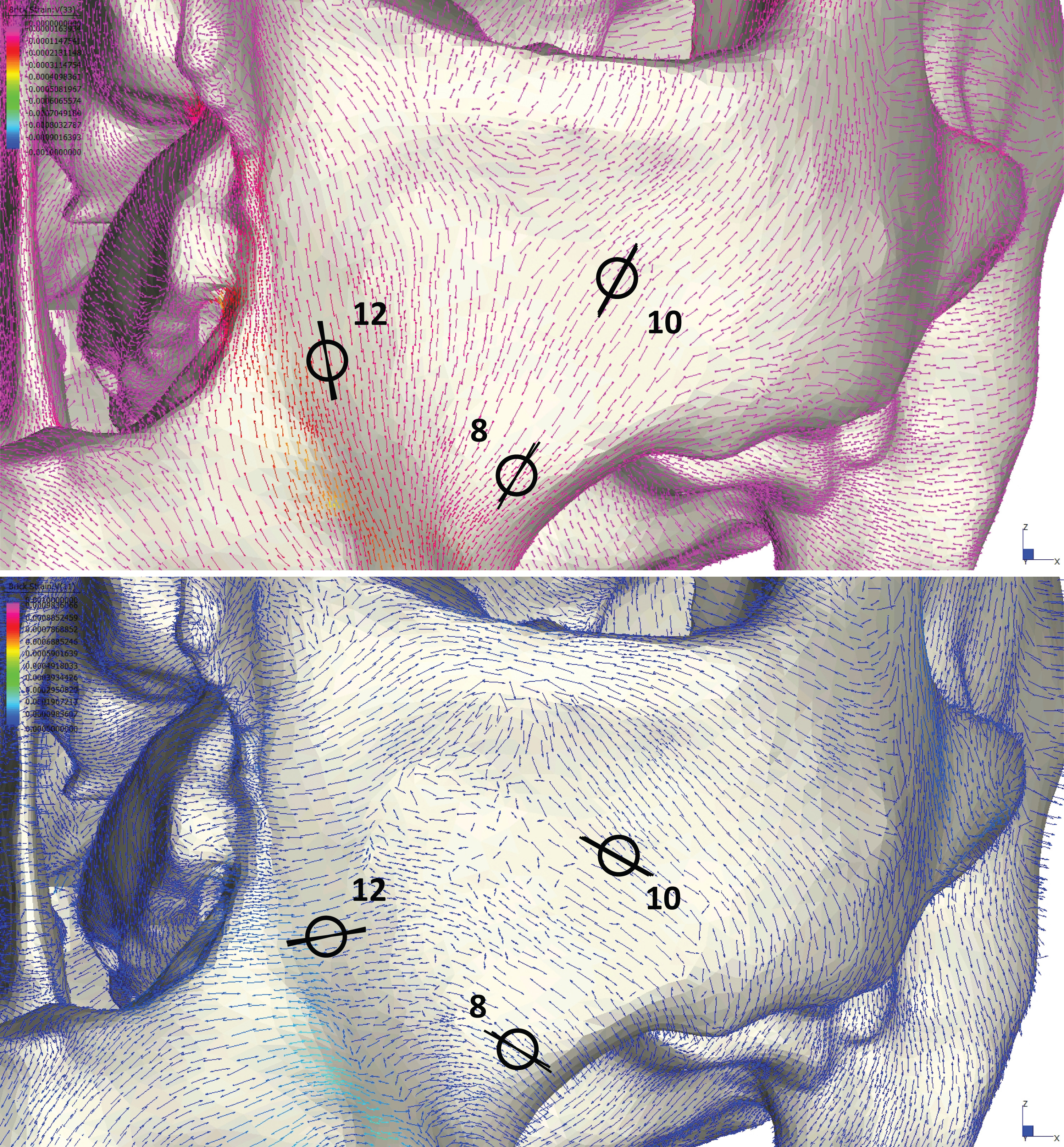

Supplement: Supplemental Information 9 — Blue lines represent the orientation of maximum principal strain (tension). Purple lines represent the orientation of minimum principal strain (compression), which is 90° to orientation of maximum principal strain. Black circles represent location of strain gages at the working-side zygomatic root (site 8), working-side infraorbital (site 10), and working-side nasal margin (site 12) during in vitro bone strain analysis. Three lines through each gage correspond to the orientation of principal strains during the in vitro loading analysis which were recorded in degrees relative to the A element of the gage. [file peerj-04-2242-s009.jpg]

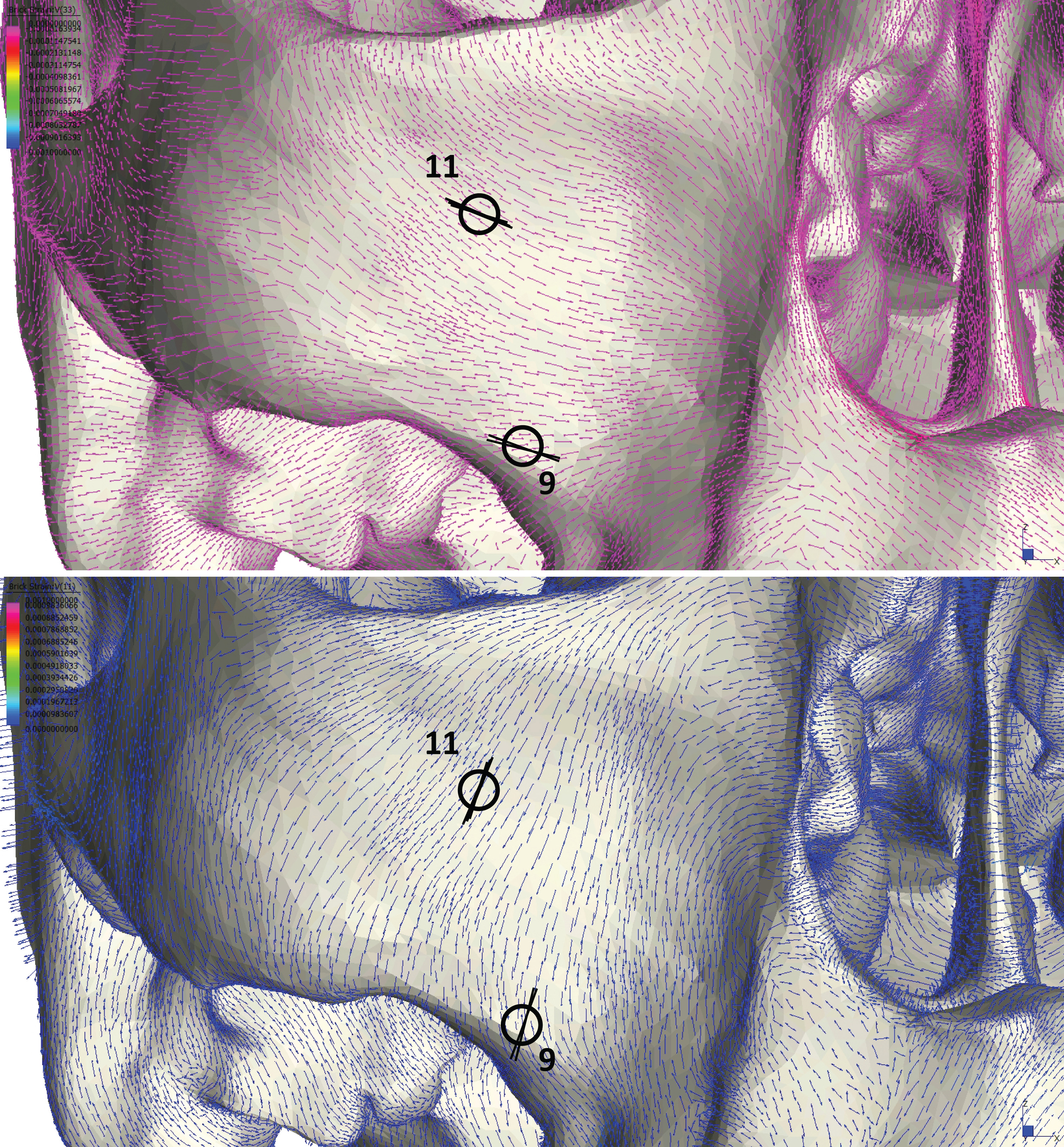

Supplement: Supplemental Information 10 — Blue lines represent the orientation of maximum principal strain (tension). Purple lines represent the orientation of minimum principal strain (compression), which is 90° to orientation of maximum principal strain. Black circles represent location of strain gages at the balancing-side zygomatic root (site 9) and balancing-side infraorbital (site 11) during in vitro bone strain analysis. Three lines through each gage correspond to the orientation of principal strains during the in vitro loading analysis which were recorded in degrees relative to the A element of the gage. [file peerj-04-2242-s010.jpg]

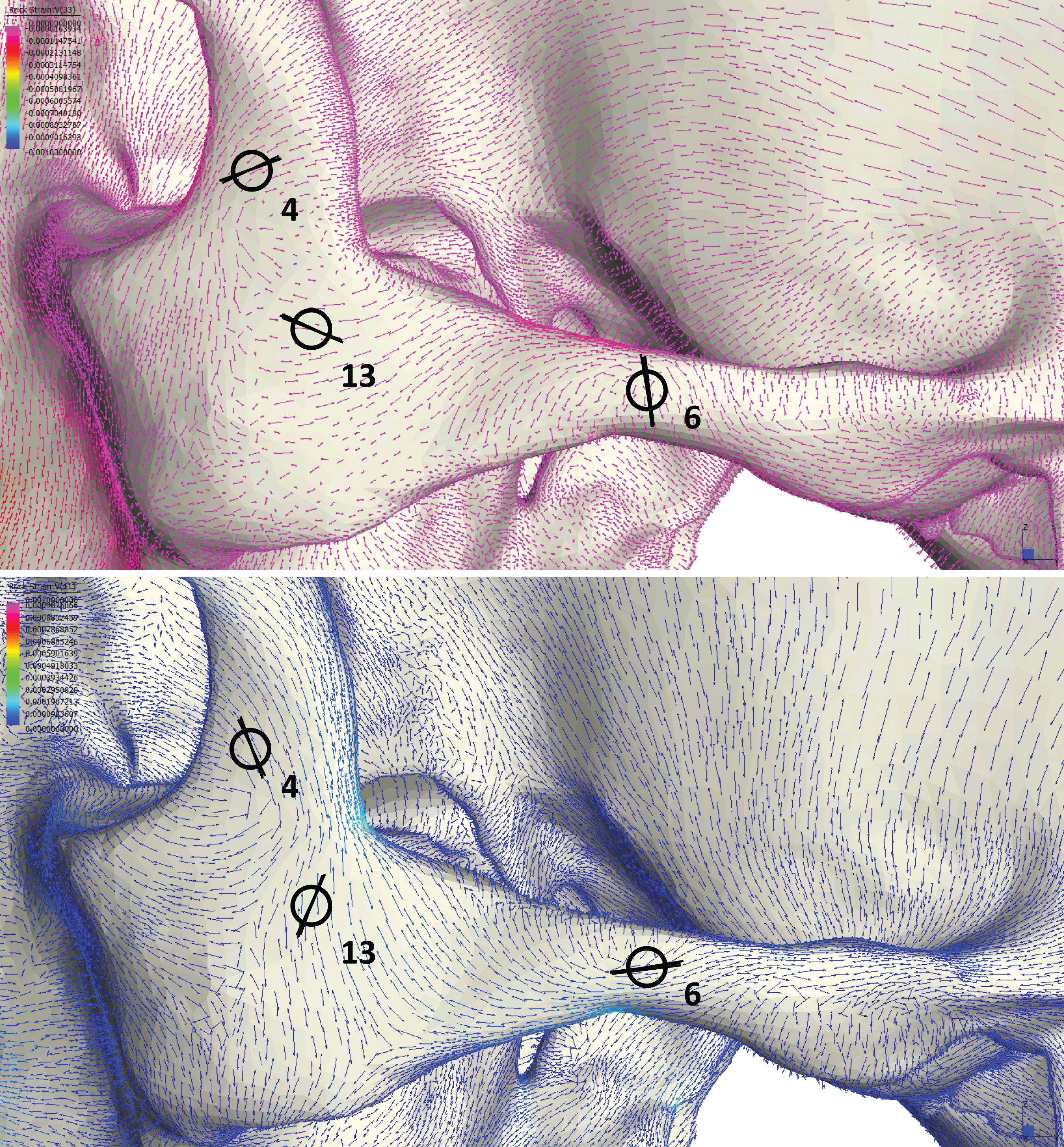

Supplement: Supplemental Information 11 — Blue lines represent the orientation of maximum principal strain (tension). Purple lines represent the orientation of minimum principal strain (compression), which is 90° to orientation of maximum principal strain. Black circles represent location of strain gages at the working-side postorbital bar (site 4), working-side zygomatic arch (site 6), and the working-side zygomatic body (site 13) during in vitro bone strain analysis. Three lines through each gage correspond to the orientation of principal strains during the in vitro loading analysis which were recorded in degrees relative to the A element of the gage. [file peerj-04-2242-s011.jpg]

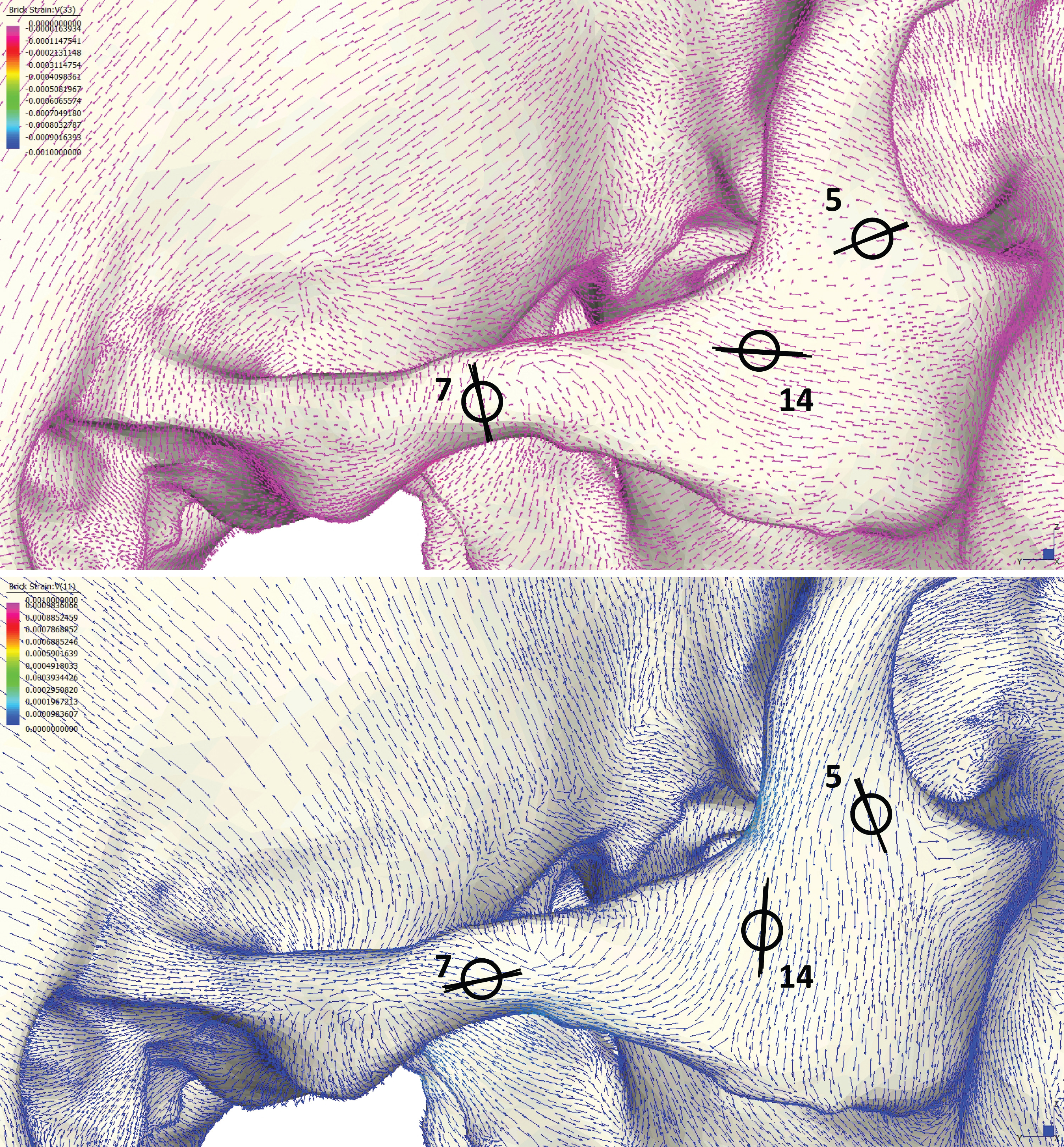

Supplement: Supplemental Information 12 — Blue lines represent the orientation of maximum principal strain (tension). Purple lines represent the orientation of minimum principal strain (compression), which is 90° to orientation of maximum principal strain. Black circles represent location of strain gages at the balancing-side postorbital bar (site 5), balancing-side zygomatic arch (site 7), and balancing-side zygomatic body (site 14) during in vitro bone strain analysis. Three lines through each gage correspond to the orientation of principal strains during the in vitro loading analysis which were recorded in degrees relative to the A element of the gage. [file peerj-04-2242-s012.jpg]

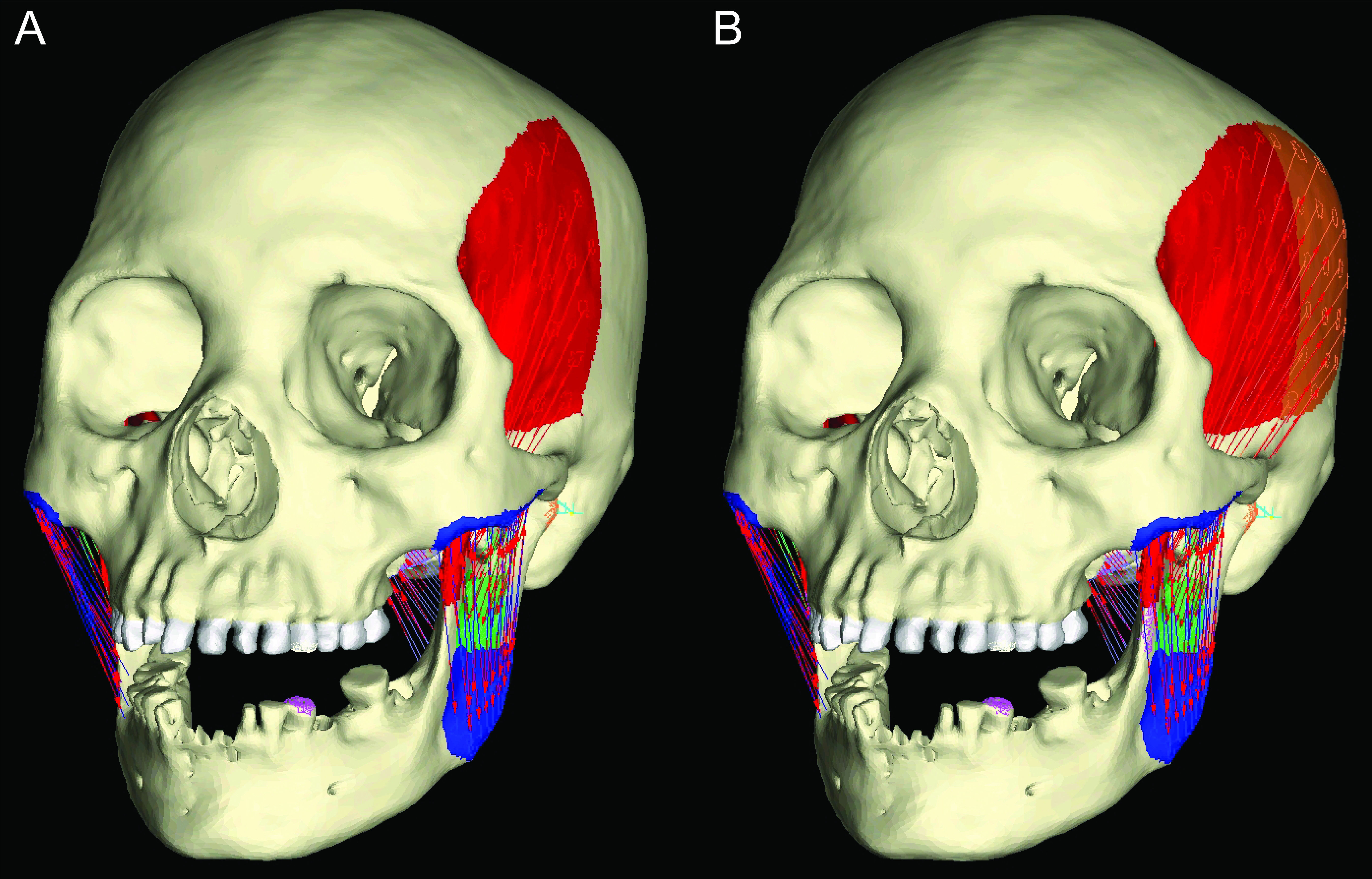

Supplement: Supplemental Information 13 — We compared two variants of this “beamed” model to our original “boneloaded” model, one that only included muscle beams for the anterior temporalis, superficial masseter, deep masseter, and medial pterygoid muscles (A) and a second that also included that posterior temporalis (B). [file peerj-04-2242-s013.jpg]

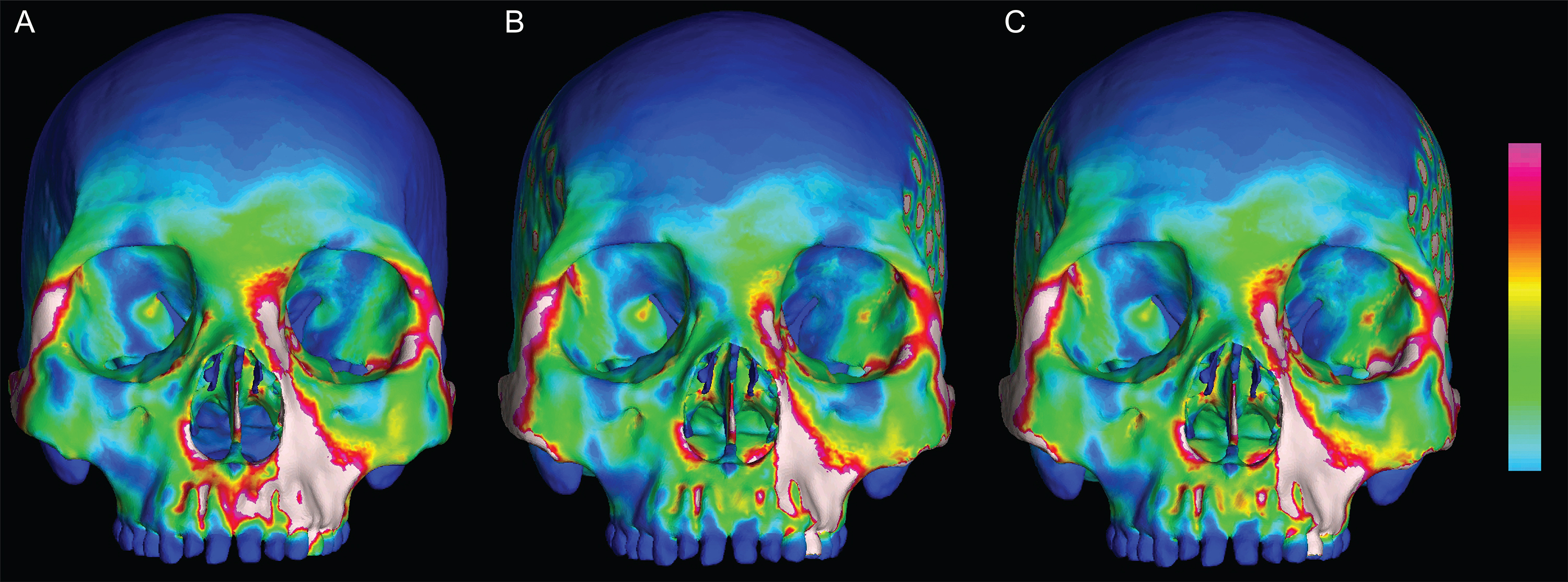

Supplement: Supplemental Information 14 — Panels show strain distributions during premolar (P3) biting in the (A) original “boneloaded” ALL-HUM model, (B) “beamed” model lacking a posterior temporalis, and (C) “beamed” model including a posterior temporalis. Scales are set to range from 0–300 μɛ White regions exceed scale. [file peerj-04-2242-s014.jpg]
